# An infant with omenn syndrome: A case report

**DOI:** 10.1016/j.amsu.2022.103319

**Published:** 2022-01-28

**Authors:** Ubaid Khan, Rana Uzair Ahmad, Ayesha Aslam

**Affiliations:** a Department of Medicine, King Edward Medical University Lahore, Pakistan; b Department of Neurology, Mayo Hospital Lahore, Pakistan

**Keywords:** Omenn syndrome, Severe combined immuno-deficiency syndrome, Autosomal recessive, Genetic mutation, Drug resistance

## Abstract

**Background:**

Omenn syndrome is a rare, autosomal recessive disorder. It is a disorder that stems from severe combined immunodeficiency in an affected individual. The disease is also of rare occurrence in children in Pakistan because this is the age where this disease usually presents, after having developed in the child. This case report will explore all the reasons that lead to the occurrence of this syndrome while also reflecting on the management profile of the child.

**Case presentation:**

This case report deals with the discussion of one such child who was diagnosed with Omenn Syndrome after being diagnosed with several other diseases, which were being mistaken for being the actual problem and disease, but the reality was that the child was suffering from Omenn syndrome.

**Discussion:**

The child is only 3 months old and his symptoms pose a great risk to his overall health status while also making him predisposed to develop several complications and nutritional deficiencies as a result, all of which keep on adding to the burden of diseases that the child faced. In Omenn syndrome, there is an increased susceptibility to develop infections of the skin, lungs, joints, as well as sepsis. Usually, the death of the patient ensues due to pneumonia and septicemia or septic shock.

**Conclusion:**

Omenn syndrome is a rare disease caused by missense mutations in the recombinase activating genes. It can be treated by bone marrow transplantation or lymphocytic stimulation depending on the severity of the presenting underlying immunodeficiency.

## Highlights

• Omenn syndrome is severe combined immune deficiency syndrome which is rare in children.
• Omenn syndrome are caused by missense mutations in the recombinase activating genes (RAG-1 and RAG-2). Mutations in these genes cause dysregulation in the B and T-cell functions.
• This case report deals with the discussion of one such child diagnosed with Omenn’ Syndrome who was mistakenly diagnosed with other disease, but the reality was that the child was suffering from Omenn syndrome.

## 1 Introduction

Omenn Syndrome was first described in 1965 by Omen GS. He had discovered some members of his extended American-Irish family were suffering from mixed symptoms. These symptoms comprised recurrent infections, lymphadenopathy, skin eruptions, eosinophilia, hepatosplenomegaly, and failure to thrive, with myriad of respiratory and gastrointestinal complications that vary in intensity, frequency, and severity from person to person [1].

Since the patient is rendered to be immunodeficient, as is obvious from his conditions, there is a high risk for him to get affected by ensuing infections which complicate the present picture and make the patient suffer even more due to these infections. If the condition is not controlled, diagnosed, and treated properly on time, then there is a high probability for the condition to turn lethal, proving to be fatal for the child [2].

Omenn syndrome has been identified to be a heterogeneous condition. Unidentified genetic defects may be present in affected individuals, which might be difficult to diagnose, thus further delaying the diagnostic process.

To date, the most common genes that have been found in patients suffering from Omenn syndrome are missense mutations in the recombinase activating genes (RAG-1 and RAG-2). Mutations in these genes cause dysregulation in the B and T-cell functions [3].

The same was the condition of the patient who has been discussed in this clinical case report. The child was male and his age was 3 months 22 days when his initial course of the disease began, as a general high-grade fever with presenting symptoms resembling a respiratory-illness like disease. Unfortunately, it was too late till the child's disease got diagnosed and he succumbed to his disease-related complications. Later, his previously collected samples, along with those of his parents and only sibling, an elder brother, were sent for genetic analysis of the genetic mutation that might otherwise have caused the disease.

All the results, management plans, and the other details that led to the final diagnosis of the patient, which were unfortunately too late to save the life of the child, have been elaborated and discussed in the preceding section. This work has been reported in line with the SCARE 2020 Guidelines [4].

## 2 Case report

A male infant, aged 3 months 22 days, weighing 6.3 kgs, was brought to the OPD on July 27, 2020 with complaints of high-grade fever, cough, noisy chest, irritability, and a generalized feeling of unwellness as reported by the parents since the last five days.

Looking at the age of the patient, the presenting complaints, and the associated symptoms, the initial diagnosis that was suspected was bronchial pneumonia with probable sepsis.

To confirm the diagnosis, the parents of the child were ordered to get his blood and urine cultures done. Both the reports came showing an obvious growth of Enterococcus.

The child was immediately started on a course of intramuscular antibiotics, including injection Linezolid, injection Amikacin, and injection Claforan. Despite receiving these injections, the child was febrile and the fever was still high grade. Therefore, he was again sent for a blood culture to rule out underlying sepsis, if any were present. A lumbar puncture (LP) was ordered this time as well. The findings of both these investigations came out to be unremarkable. Knowing that the child had no further progression of the disease, as seen from his lab reports, it was decided that the antibiotic injections would be continued as usual from the first time.

Apart from that, new injections, including injection Vancomycin and injection Meropenem were started to ward off any underlying infection. But again, the child fever was persistently present and it was still in the high-grade category.

Now, it was decided that the patient should be started on Azomax. But before starting Azomax, it was decided that both the injections of Vancomycin and Meornem would be stopped and only Azomax would be continued until the resolution of the infection. However, this time a new development was seen. The child developed fits while still having fever. There were two reported episodes of fits with fever and also, the child was now unable to maintain oxygen at room air level.

While starting the child at a constant infusion of 1 L O2, his third blood culture was set for further evaluations and progressions. The blood culture was still showing a Gram-positive culture growth. Meanwhile, the child's bone marrow biopsy and culture were also scheduled to be performed within two weeks from the current date. His bone marrow aspiration was, however, done in the meantime.

This bone marrow aspiration report revealed that the child was suffering from Hemophagocytic Lymphohistiocytosis (HLH) Syndrome.

This syndrome is a potentially life-threatening and aggressive condition in which there is excessive, unwanted activation of the immune system. It is most prevalent in infants up to the age of 18 months, but adults could be affected too [5].

As of the last written notes in the patient's files, his bone marrow biopsy and culture reports were awaited to date.

● ***Investigations***

Since the condition of the child kept on deteriorating and getting severe with time, therefore it was decided that his investigations would be constantly kept in the loop to assess his condition and check for any progressions or regressions in his lab reports.

Therefore, in a summarized form, here is an overview of all the investigations that were reported for the child along with the comments on them.

**Table:** 

WORKUP/INVESTIGATIONS	COMMENTS
Plasma Ammonia	31 (Normal)
Plasma Amino Acids	Sent to AKU Labs for analysis
Urine Organic Acid Test	Sent to AKU Labs for analysis
C-Reactive Protein	Normal throughout admission
ECHO	Normal
Immunoglobulins	● **IgM:** 0.3 (Decreased) ● **IgA:** Normal ● **IgE:** Normal ● **IgG:** Normal
Total + Ionized Calcium	Normal
Chest X-RAY	Normal
U/S Abdomen	Normal
U/S Skull	Normal

● ***Management Course:***

The summary of the drug history that was administered to the patient until the last time is summarized as follows:

**Table:** 

NAME	COMMENTS
Inj. Vancomycin	12 days course completed
Inj. Meronem	11 days course completed
Inj. Azomax	9 days course completed
Pentaglobin	3 doses administered to date
Inj. Gen-M	Course completed
Inj. Decadron	Was just recently started

Throughout the management course, his vitals were hourly measured. Despite the timely administration of the child antibiotic doses, his fever kept on waxing and waning and it never touched to baseline.

This child soon succumbed to his prevailing conditions and was unable to make it through. As devastating as this was for the parents, there were still some samples left of the child, which could serve as an important means for detecting if there was any other abnormality or even a genetic mutation that made diagnosis and treatment so difficult. The child was scheduled for chemotherapy, to stabilize the patient for bone marrow transplant. The death of child occurred during third cycle of chemotherapy.

Therefore, not only the child samples, but also those of his parents, who were involved in a consanguineous marriage, and also that of the child elder brother were sent for a genetic analysis, which would then detect whatever was wrong within his body.

Sanger sequencing was used to check for RAG1 gene mutation, which are detected in people suffering Omenn syndrome. The elder brother of the child was tested clear and has no active gene or the carrier gene for this mutation. The father of the child was found to possess a carrier gene for this pathogenic mutation, whereas the mother of the child was also found to possess the same carrier gene.

This report was sufficient enough to confirm that the deceased child was indeed suffering from Omenn syndrome that had been passed on to him from the pathogenic variant that was present in both his parents and had ultimately resulted in his death.

## 3 Discussion

Omenn syndrome is a rare genetic disorder that is inherited in an autosomal recessive pattern in infants and young children. In this disease, there is severe combined immunodeficiency that involves multiple organs and systems of the affected children. The affected children might present with a myriad of symptoms including lymphadenopathy, hepatosplenomegaly, eosinophilia, erythroderma, increased or recurrent infections, poor growth, and increased serum IgE levels. If a child presents with skin lesions, they could easily mimic to be like those that appear in graft-versus host disease [6].

All cases of Omenn syndrome are seen to have a fatal outcome within two months unless stem cell transplantation done in these patients [7].

However, these patients are usually diagnosed lately and this lead to their earth because of the fatal, ensuing complications. In some of the cases, where even the child could survive beyond two months and was getting his transplantation finalized, there would be some complications that would prevent this transplant from taking place [8].

In Omenn syndrome, there is an increased susceptibility to develop infections of the skin, lungs, joints, as well as sepsis. Usually, the death of the patient ensues due to pneumonia and septicemia or septic shock [9].

The major cause of this disease is a missense mutation in the recombinase activating genes, RAG-1, and RAG-2. These mutations prevent normal maturation of B and T cells. This leads to a depletion in the levels of lymphocytes in the thymus and lymphoid tissues of the affected individuals [10].

For the treatment of Omenn syndrome, either lymphocyte stimulation tests or bone marrow transplantation are done to treat the presenting primary immunodeficiency. Similarly, genetic analysis of the immediate family members of the affected individual should be done at the same time to see if the siblings are also at the risk of developing this disease sometime later so that preventive measures could be taken to avoid the condition once and for all [11].

## 4 Conclusion

Omenn syndrome is severe combined immuno-deficiency syndrome due missense mutations in the recombinase activating genes (RAG-1 and RAG-2). Mutations in these genes cause dysregulation in the B and T-cell functions. Its rare in children. Presentation is variabvle in children. Hemophagocytic Lymphohistiocytosis (HLH) Syndrome. It can be treated by bone marrow transplantation or lymphocytic stimulation depending on the severity of the presenting underlying immunodeficiency.

## Consent

Written informed consent was obtained from the parent of patient for publication of this case report. A copy of the written consent is available for review by the Editor-in-Chief of this journal on request.

## Guarantor

Ubaid Khan.

## Funding sources

None.

## Ethical approval

For this case ethical approval was not required from hospital, and we have patient father consent form.

## Registration of research studies

As this is case report the registration not required.

## Provenance and peer review

Not commissioned, externally peer reviewed.

## Author's contribution

All authors contributed toward data analysis, drafting, and revising the paper, gave final approval of the version to be published, and agree to be accountable for all aspects of the work.

## Declaration of competing interest

Ubaid Khan, Rana Uzair Ahmad and Ayesha Aslam declare that they have no conflict of interest in this publication.
